# A Treatise on Uterine Hæmorrhage

**Published:** 1817-04

**Authors:** 


*A Treatise on Uterine Hæmorrhage.* By Duncan Stewart, Physician Accoucheur to the Westminster General Dispensary, and Lecturer on Midwifery in London. 8vo. pp. 151.

It is a general fault among persons who write on practical subjects, that they fritter away time in useless conjecture, and indulge in hypothetical reasonings upon matters which really serve no good purpose; and which, in point of utility, stand in direct contradiction to the author's avowed object.

The laws which regulate the muscular actions of animal bodies, as well as those which lead to the development of disease, are so recondite as to set at defiance the best directed efforts of human sagacity. Were the mind of man, however, capable of even demonstrating them, "it is," to use our author's words, "very probable that a knowledge of them would not add to our stock of useful information."

The introductory chapter of this work chiefly consists of observations upon the elastic and muscular powers of the uterus; and although nothing new is elicited, it is satisfactory to find, that there is no attempt to take the reader into the regions of fancy, nor to give to airy nothings a local habitation and a name.

The author divides his book into four sections.

SECTION 1. contains Observations on the Practice generally employed in Uterine Hæmorrhage.

The mode of practice now generally and successfully employed by practitioners in midwifery, when hæmorrhage takes place, anterior to, as well as co-existent with the full period of utero-gestation, is here objected to, notwithstanding the principle of emptying the uterus by puncturing the membranes is admitted. The author considers that this measure is too frequently resorted to, without a due regard to the patient's safety; that fearful and indolent practitioners find an excuse in it, by flattering themselves that they have done all that can be done; and that the practice is often attended with fatal consequences.

Before rupturing the membranes, the three following questions are proposed to be maturely weighed:—1. Whether, by puncturing the membranes before the os uteri is dilated, the delivery of the child is retarded or accelerated? 2d. Whether we can depend upon it as a certain and immediate means of suppressing the hæmorrhage? 3. Whether, by puncturing the membranes before the os uteri is dilated, we do not often destroy the chance of saving the child's, and sometimes even the mother's life.

The author embraces the negative side of the question in his observations on the 1st query, for reasons which do not appear to us sufficiently cogent: viz. because *the uterus does not always contract immediately*; because *that contraction cannot be pronounced upon with certainty*; and because *protracted labour is the general result of an evacuation of the liquor amnii.*

The observations which we have had occasion to make upon the practice of rupturing the membranes, confirm an opinion which stands at variance with that given above.

The following are the author's own words upon the two latter questions:

"In answering the second query, whether the puncturing the membranes acts as a certain means in suppressing uterine hæmorrhage, two positions come to be considered:—1st. Whether it allows the uterus to contract so as materially to lessen the size of the ruptured vessels? And 2dly, Whether, supposing the uterus to contract immediately after the evacuation of the liquor amnii, such a degree of forcible pressure will be made on the mouths of the ruptured vessels as to suppress the hæmorrhage. Any diminution that can take place in the size of the uterine vessels by the mere discharge of the liquor amnii, must be trifling; and as the quantity of that fluid varies from half a pint to several quarts, it will be impossible, in any case, to form a correct judgement of the degree of contraction which may be expected to take place by puncturing the membranes. But, even granting that the uterus, by contracting, considerably diminishes the diameter of its vessels, as long as the child is alive, and still in the uterus, the fœtal mode of life will be continued; and in this respect, the functions of the organ will remain unaltered: the quantity of blood circulating towards its vessels will, therefore, continue undiminished, their action will be necessarily increased, and the hæmorrhage will continue unabated. In this view of the subject then, the evacuation of the liquor amnii can be of little service in restraining uterine hæmorrhage. If, after the evacuation of the liquor amnii, the uterus should immediately contract, and bring that portion of the placenta which is detached, to press so forcibly against a level surface on the child's body, that a degree of firm and constant pressure is made on the mouths of the bleeding vessels, no doubt the discharge will be stopped. But it has already been shewn that it is not always certain that the uterus will be immediately excited to contraction by the discharge of the liquor amnii. Those who most strenuously advise the early puncturing of the membranes, admit that it sometimes fails in checking the discharge. If the membranes are ruptured before the os uteri is dilated, we must be at the time ignorant both of the child's position, and of the situation of the placenta, if it does not present over the os uteri. When the hæmorrhage, therefore, is checked in consequence of the detached portion of the placenta being brought to press against the child's body, it must evidently be, in a great measure, the effect of chance.

"Although the uterus, whilst vigorous, possesses the power of containing itself in a contracted state, yet it is only during the expulsive efforts that the child's body is so closely embraced, as, by pressure, to check the hæmorrhage. During the remission of these efforts, therefore, the discharge will return; and if the mouths of the bleeding vessels are not shut up by pressure, whilst the uterus is exerting its expulsive efforts, at this time the action of the heart and arteries being greatly increased, the discharge will be very profuse.

"It may, therefore, be concluded, that, as a general rule of practice in uterine hæmorrhage proceeding from accidental detachment of the placenta, the plan of rupturing the membranes before the os uteri is completely dilated, *is neither safe nor rational.*"

Upon a subject of such vital importance as the present, it is presumed that no person would take upon himself to decry a mode of practice without substituting a better in its place. Long experience in cases of uterine hæmorrhage has taught us how to appreciate the practice of diminishing the bulk of the uterus, by letting off the aqueous contents; and, until we see more solid arguments against its utility, and less success in its adoption, we must dissent from the opinion of our author.

The observations on the practice generally recommended in uterine hæmorrhage from detention of the placenta are common place, and propose nothing that was not before well understood.

Section 2 comprises the author's mode of treatment, when hæmorrhage occurs in the early months of pregnancy. Although there are few or no respectable practitioners in the obstetric art who are not acquainted with the routine of practice, yet, as the younger and inexperienced cannot have the impression too firmly graven upon the mind, and as this affords a fair specimen of the author's practical notions, we shall transcribe it.

"As in a healthy state, no discharge of blood comes from the uterus during the period of pregnancy, every discharge of blood during this period, however trifling, demands attention.

"Uterine hæmorrhage may take place at any period from the time of impregnation, till many hours after the delivery of the child and secundines at the full period of pregnancy; and as there is generally a very considerable difference in the causes which produce the disease, in its symptoms, in its termination, and in the plan of treatment which should be adopted, according to the period at which it takes place, it is necessary to consider it First, as it occurs previous to the sixth month of pregnancy; secondly, as it occurs in the last month of pregnancy; and, during the process of parturition at the full period of pregnancy, till the child is delivered: and, thirdly, as it occurs immediately before and after the placenta is expelled.

"The ovum is connected to the uterus solely by means of vessels; and any cause which produces a considerable degree of derangement of the general system, or which impedes the due function of the uterine system, is apt in part to destroy the connexion, and to cause a discharge of blood. This accident is particularly apt to take place at the periods at which a woman should have menstruated, had she not become pregnant. As the quantity of blood discharged in these cases will be in proportion to the extent to which the ovum is separated from the uterus, it may be trifling, and only take place when any exertion is made. It is often accompanied with pain in the back; or it may be considerable, and unaccompanied by pain; or it may be profuse either with or without pain. Considerable constitutional derangement, with a state of great irritability of the general system and fits of hysteria, sometimes accompany the discharge. When the hæmorrhage is not very profuse, by confining the patient to a horizontal posture, keeping her in a good temperature, bleeding her if she is of a plethoric habit, allaying irritation by opiates, keeping the bowels regular, adhering strictly to the antiphlogistic plan, and applying cold water frequently to the back, pubes, and vulva, the discharge will generally disappear. But if, notwithstanding the use of these means, the hæmorrhage should continue, considerable advantage may be derived from injecting some cold astringent fluid into the vagina; or from giving a cold clyster, consisting of one pound of lime-water and one or two drachms of laudanum. Sometimes all these remedies will prove unavailing, the discharge still continuing, and even increasing; a proof that a great part, or the whole of the ovum, perhaps, is detached from the uterus, and that abortion will inevitably take place. In some cases, although the discharge disappears, it recurs at irregular intervals, and in considerable quantity, till at the fourth or fifth month, or perhaps at a later period of pregnancy, an ovum of two or three months growth is voided. In these cases, confinement to a horizontal posture, injecting some cold astringent fluid into the vagina, applying cold water to the back and pubes, and giving, two or three times a day, small doses of sulphuric acid, will generally be found to check the discharge till the ovum escapes from the uterus. The strength may likewise be supported by moderate quantities of light wine.

"Although abortions are frequently tedious, and often accompanied with a very considerable discharge of blood, they seldom prove fatal from this cause alone, if properly treated. It is seldom necessary or practicable to give manual assistance in the early months of pregnancy; and although in some cases on record, it is stated, that the hand was introduced into the uterus at the fifth month, the practice cannot be justified; as at this early period, when the parts are as yet in a very contracted state, it must do great injury. When abortion is attended with very profuse hæmorrhage, the discharge can be easily commanded by stuffing the vagina. When this remedy is employed, it is necessary to fill the vagina completely, and to avoid as much as possible every thing that will cause pain and irritation. Soft rag, or lint, soaked in oil, is to be cautiously introduced into the vagina; and a firm compress, wet with cold water, is to be fixed by a T bandage over the external orifice, so as to prevent the plug from being displaced. By this means a coagulum is allowed to form at the mouths of the bleeding vessels, which prevents any further discharge of blood. It will likewise be necessary to confine the patient to a horizontal posture, to apply cold to the thighs and pubes, to admit cool air freely into the bed-chamber, and to avoid the use of stimulants.

"As a complete state of contraction of the uterus is the only means which can permanently stop a profuse hæmorrhage from that organ, it will be also necessary, whilst remedies are employed to moderate the discharge of blood, to excite the uterus to contract, in order to expel its contents; for this purpose stimulating clysters will be found very beneficial: but when they are given, the practitioner ought always to be in attendance, as, if the plug be displaced, a sudden and alarming increase of the hæmorrhage may occur. When the uterus begins to contract frequently and powerfully, the bandage and compress may be removed; but the plug should be allowed to remain till it is expelled, as it will keep up a degree of irritation on the os uteri, which will tend to increase and continue the expulsive efforts of the uterus, and, by retarding in some degree the expulsion of the ovum, it will also favour the complete contraction of the uterus.

"Frequent fits of syncope are apt to accompany abortions, although there has been no previous great discharge of blood; but this symptom generally proceeds more from an affection of the nervous system than from debility. If the discharge of blood, however, has been very profuse, the strength reduced, and symptoms of great debility supervene, whilst means are employed to suppress the discharge, stimulants, in moderate quantity, must be also given to support the strength. Opium in large doses, either in a solid or liquid form, seems best calculated for this purpose; for whilst it quiets the anxiety which the patient's apprehensions are apt to produce, it allays the state of general irritation, which always attends a sudden diminution of the powers of life, supports the strength without accelerating the action of the heart and arteries, and never prevents the due contraction of the uterus."

A case follows, illustrating the foregoing remarks.

SECTION 3. The introductory remarks in this division of the book are to the purpose; but the practice does not accord either with the opinion of the best writers and practitioners of this day, nor with the judgement that ample opportunity has enabled us to form. The lessening of the capacity of the uterus, when hæmorrhage to a great extent takes place by rupturing the membranes, has always been followed by an interruption to the discharge; and when pains have followed, we have never had any apprehension for the future welfare of our patient, notwithstanding the os uteri may remain rigid and unyielding for many hours. This *perplexing occurrence*, as the author terms it (vide page 70), has satisfied us as to the prospect of events; on the contrary, we have regarded, as an unpleasant occurrence, the flaccid condition of this part, the degree of which is an admirable test for manual interference, as well as an indication of extreme prostration, and of the loss of vital energy on the part of the uterus itself.

SECTION 4. On the causes and treatment of uterine hæmorrhage, when it occurs after the child is delivered.

This division of the work is worth the rest of the book: it shews, in perspicuous and expressive language; a rational mode of practice. We select the following:

"In patients who have had attacks of uterine hæmorrhage after former labours, from a state of torpor of the uterus; or when, from their general appearance, or other circumstances, a state of torpor of this viscus is to be dreaded at this time; a broad bandage should be applied over the abdomen as soon as the labour commences, which should be gradually tightened as the process advances. This will support the uterus, and assist it in retaining a contracted state. When the labour is natural, and advancing, though slowly, every mean which would tend to fatigue or irritate the patient should be avoided, and the child should be allowed to be completely expelled by the natural efforts. As soon as the child is delivered, the bandage should be drawn as tight as the patient can bear it, without rendering her uncomfortable. The application of the bandage will not only support the uterus, but will be found to obviate a strong tendency to syncope, which generally produces a state of torpor of that viscus, and which so frequently attends the sudden removal of an accustomed pressure from distended parts. As soon as all dread of hæmorrhage is removed, the bandage may be gradually removed.

"In hæmorrhage proceeding from torpor of the uterus, when the discharge of blood is very great, in addition to the application of the bandage, one hundred drops of laudanum should be given; and the hand, well oiled, should be gently introduced into the uterus. It should be kept in mind, that the object to be accomplished, by introducing the hand, is not to bring away the placenta, but to excite the uterus to contract, that it may separate the placenta, force it into the vagina, and thus permanently check the hæmorrhage."

Nothing that falls within the province of the accoucheur makes such a lively appeal to his feelings, or calls for such unruffled composure of mind; so much skilful judgement and steadiness of manner in the exercise of it, as hæmorrhage from the uterus; whether it takes place at the time of parturition, or precedes by a few weeks that occurrence.

It has often fallen to our lot, from being extensively engaged in this department of the profession, to be present at some of the worst cases of the kind; to have to participate in joy at successful terminations, and in reverses to mingle our sighs with the tears and regrets of surrounding relatives.

We took up the present volume with feelings of pleasure, and we lay it down with satisfaction. On some points we differ from the author, but approve of the work upon the whole, as containing some excellent instructions, founded, as we perceive, upon information derived from the best of sources, experience.

On the good effects of opium, so largely exhibited and so much extolled by the author, we are not competent to judge, never having used it in the manner and to the extent he recommends. Reflecting on its operation upon the system in general, and considering also the manual interference which, in our author's practice, accompanies its use, we feel inclined to believe, that its good effects are somewhat over-rated. On this subject, however, we wish it to be understood that we require further information.

